# Modeling Biometric Traits, Yield and Nutritional and Antioxidant Properties of Seeds of Three Soybean Cultivars Through the Application of Biostimulant Containing Seaweed and Amino Acids

**DOI:** 10.3389/fpls.2018.00388

**Published:** 2018-03-27

**Authors:** Sławomir Kocira, Agnieszka Szparaga, Anna Kocira, Ewa Czerwińska, Agnieszka Wójtowicz, Urszula Bronowicka-Mielniczuk, Milan Koszel, Pavol Findura

**Affiliations:** ^1^Department of Machinery Exploitation and Production Process Management, Faculty of Production Engineering, University of Life Sciences in Lublin, Lublin, Poland; ^2^Department of Agrobiotechnology, Faculty of Mechanical Engineering, Koszalin University of Technology, Koszalin, Poland; ^3^Institute of Agricultural Sciences, State School of Higher Education in Chełm, Chełm, Poland; ^4^Department of Biomedical Engineering, Faculty of Technology and Education, Koszalin University of Technology, Koszalin, Poland; ^5^Department of Food Process Engineering, Faculty of Production Engineering, University of Life Sciences in Lublin, Lublin, Poland; ^6^Department of Applied Mathematics and Computer Sciences, Faculty of Production Engineering, University of Life Sciences in Lublin, Lublin, Poland; ^7^Department of Machines and Production Biosystems, Faculty of Engineering, Slovak University of Agriculture in Nitra, Nitra, Slovakia

**Keywords:** antioxidant activity, free amino acids, *Glycine max*, growth, nutrients, seaweed, yield

## Abstract

In recent years, attempts have been made to use preparations that allow obtaining high and good quality yields, while reducing the application of pesticides and mineral fertilizers. These include biostimulants that are safe for the natural environment and contribute to the improvement of yield size and quality, especially after the occurrence of stressors. Their use is advisable in the case of crops sensitive to such biotic stress factors like low temperatures or drought. One of these is soybean which is a very important plant from the economic viewpoint. Field experiments were established in the years 2014-2016 in a random block design in four replicates on experimental plots of 10 m^2^. Three soybean cultivars: Annushka, Mavka, and Atlanta were planted in the third decade of April. Fylloton biostimulant was used at 0.7% or 1% concentrations as single spraying (BBCH 13-15) or double spraying (BBCH 13-15, BBCH 61) in the vegetation period. The number of seeds per 1 m^2^, seed yield, thousand seed weight, number of pods per plant, number of nodes in the main shoot, height of plants, and protein and fat contents in seeds were determined. The content of phenolic compounds, antioxidant capacity and antioxidant effect of soybean seeds were assayed as well. Foliar treatment of soybean with Fylloton stimulated the growth and yield of plants without compromising their nutritional and nutraceutical properties. The double application of the higher concentration of Fylloton was favorable for the plant height, seed number and soybean yield. Moreover, the highest number of pods was obtained after single treatment of plants with the lower biostimulant concentration. There was also a positive effect of using this biostimulant on the content and activity of some bioactive compounds, such as phenolics and flavonoids, and on the reducing power.

## Introduction

The main objective of agricultural holdings is to produce the highest amount of crops, while maintaining the highest quality. This is due to the continuously increasing demand for high-quality raw materials produced with the lowest possible exposure to pesticides. The increasing occurrence of stressors (drought, frosts, high temperatures, salinity, heavy metal pollution, pests, and pathogens) is one of the main causes of the decline in crop yields. Both, too low and too high temperatures have negative effects on the growth, development and yielding of crops, especially when they occur in periods critical to the crop. In soybean ontogenesis, the first period being critical to the growth and development of its plants is the sprouting stage when seed germination is impaired by low temperature, which may eventually extend the emergence stage to even 45 days. Adverse conditions for germination facilitate plants infestation by soil pathogens, which results in the development of weak sprouts or even their lack (Jasinska and Kotecki, 2003). In turn, Schlenker and Roberts (2008) demonstrated that even short-lasting too high temperature had a negative impact on soybean seed yield. In addition, very high temperatures at the blooming stage may contribute to reduction in the potential number of seeds per plant, which was reported to have a negative effect on soybean yield (Wheeler et al., 2000). The extending period of high temperatures, i.e., from the stage of blooming and pod development till the stage of grain filling, causes a decrease in seed mass gain (Gibson and Mullen, 1996). The high temperature determines also the chemical composition of soybean seeds. As reported by Dornbos and Mullen (1992), seeds of soybean plants growing in conditions of medium water deficit and exposed to a temperature of 35°C at the seed plumpness stage contained more protein and less fat compared to seeds of plants growing at 29°C. Both, the excess and deficit of atmospheric precipitation have negative effects on plant growth as well as on seed yield and quality. Heavy atmospheric precipitation in the seed filling stage contributes to a decreased protein content and an increased fat content in soybean seeds (Vollmann et al., 2000). Although soybean is adapted to survive longer periods of draft, some critical periods occur during its development that are associated with demand for water, e.g., during germination, blooming and pod filling (Jasinska and Kotecki, 2003). Water deficit in the soil during drought contributes to seed yield decrease (Cox and Jolliff, 1986), and to an increased content of protein and decrease content of fats in soybean seeds (Dornbos and Mullen, 1992). When these conditions appear, it is justified to use a variety of preparations containing biologically-active substances, including biostimulants. According to Du Jardin (2015), biostimulants are preparations containing a substance(s) and/or microorganisms intended for use on a plant or root zone to stimulate natural processes that increase the efficiency of nutrient utilization, tolerance to abiotic stress, and qualitative characteristics of plants regardless of the nutrient content. This group includes also commercial products containing mixtures of such substances and/or microorganisms (Du Jardin, 2015). Biostimulants influence metabolic processes in the plant by stimulating the synthesis or increasing phytohormone activity, stimulating the growth of the root system and improving the uptake, translocation and utilization of nutrients, which determines the quantity and quality of the yield, for example, the coloring and chemical composition of the crop. Increasing plant resistance to stressors through the use of biostimulants is likely due to changes in enzymatic activity and increased synthesis of antioxidative compounds (Basak, 2008; Calvo et al., 2014).

Biostimulants may be of natural or synthetic origin and contain both organic and inorganic compounds. Natural biostimulants are based on free amino acids, humic compounds, fruit or seaweed extracts, chitin and its derivative—chitosan, or effective microorganisms. In the group of synthetic biostimulants, there are preparations containing growth regulators, phenolic compounds, inorganic salts and beneficial nutrients (Al, Co, Na, Se, Ti, Si) (Calvo et al., 2014; Przybysz et al., 2014; Du Jardin, 2015). Fylloton belongs to the natural biostimulants, and contains *Ascophyllum nodosum* seaweed extract and free amino acids obtained by enzymatic hydrolysis. Active components of this biostimulant facilitate the growth and development of plants, thereby improving both the size and quality of crop yield. Extracts form marine algae have been demonstrated to induce multiple physiological processes in plants even at low concentrations, thus contributing to their enhanced growth and blooming, to the improved size and quality of their yield and their nutritive value owing to the presence of phytohormones and low-molecular-weight compounds (Stirk and Van Staden, 1997; Tarakhovskaya et al., 2007; Battacharya et al., 2015). Equally important are polysaccharides and polyphenols present in the extract due to their allelochemical potential and capability to increase plant resistance to stress conditions (Klarzynski et al., 2003; Zhang et al., 2006; Rioux et al., 2007). In turn, amino acids—being constituents of proteins—play an important role as precursors or activators of phytohormones or take part in the synthesis of such organic compounds as: amines, purines, pyrimidines, alkaloids, vitamins, enzymes, and terpenes (Marschner, 1995; Pratelli and Pilot, 2007). They are also crucial to the process of pollination and fruit development (Stitt et al., 2002). The use of exogenous amino acids that are active in metabolic signalization (e.g., glutamate, histidine, proline, glycine, and betaine) has been demonstrated to activate defense mechanism of plants, which results in their increased resistance to abiotic stress factors (drought, salinity, extreme temperature, oxidative stress; Calvo et al., 2014; Colla et al., 2015).

Furthermore, the use of biostimulants seems justified in the case of crops that are particularly sensitive to adverse climatic conditions and are economically important. This group of plants includes, among others, the *Fabaceae* that include species sensitive to low-temperature like soybean, which is one of the most important food and feed crops.

So far, scarce studies have addressed the effects of preparations based on seaweed extracts and free amino acids on growth, development and yield of plants (Norouzpour and Abad, 2013; Kocira et al., 2015a). In addition, the changing climate causes more frequent occurrence of stressful conditions for plants, e.g., low temperatures and drought. Therefore, with the growing popularity of soybean crops, it seems reasonable to study the effect of a biostimulant based on the *Ascophyllum nodosum* extract and free amino acids on plant growth, yield soybean *Glycine max* (L.) Merr. The following research hypothesis was verified in this study: foliar application of a Fylloton biostimulant is a factor which determines plant growth, yield size, and nutritional and nutraceutical properties of seeds of three soybean cultivars (Annushka, Mavka, Atlanta).

The Annushka cultivar was chosen for the study owing to its short growing season. It is also a good fore-crop for successive crops and a plant suitable for re-sowing, e.g., after frozen stands of winter crops. In turn, Mavka cultivar is recommended for its stable ripening and high resistance to grain shedding and lodging. Finally, Atlanta cultivar is valued for a high protein content of its seeds, which is of high significance to the feed and food industries.

## Materials and methods

### Plant materials and growth conditions

Biological experimental material consisted of three cultivars of soybean *Glycine max* (L.) Merr.: Annushka, Mavka, and Atlanta (Agroyoumis, Poland). The agronomic differences between the studied soybean cultivars are shown in Table 1. Field experiments were conducted in the years 2014-2016 on the experimental plots of the Institute of Agricultural Sciences of the State School of Higher Education in Chelm, leased in a farm located in Perespa (50°66′N, 23°63′E) in Poland. The experiments were established in a randomized block design in four replicates on experimental plots of 10 m^2^ (3.00 × 3.33 m). Soybean was cultivated on the soil belonging to the brown rendzina sub-type, characterized by alkaline pH (pH in 1 M KCl−7.4–7.5). The soil content in the assimilable nutrients was as follows: phosphorus – medium (12.6–14.2 mg P_2_O_5_ in 100 g soil), potassium—medium (15.3–17.1 mg K_2_O in 100 g soil), and magnesium—medium (6.2–6.8 mg Mg in 100 g soil). Each year, winter wheat was used as a forecrop for soybean. The soil was treated with a passive combined cultivator consisting of a cultivator with teeth ending with plowshares and a double stranded shaft. Seeds of three cultivars: Annushka, Mavka, and Atlanta were sown with a mechanical precision seed drill on the following dates: April 25, 2014, April 25, 2015, and April 23, 2016. Seeds were sown in rows every 30 cm at a row spacing of 3.5 cm. No pesticides were used (pests did not exceed the thresholds of harmfulness). Biostimulant was used according to the scheme during the vegetation period (Table 2), and the results were compared with the control combination where pure water was used for spraying.

**Table 1:** The agronomic differences between the studied soybean cultivars (http://www.farmer.pl/produkcja-roslinna/inne-uprawy/odmiany-hodowli-soi-agroyoumis-polska,59844.htm).

**Phenotypic features**	**Soybean cultivars**		
	**Annushka**	**Mavka**	**Atlanta**
Height	80–110 cm	80–110 cm	100–130 cm
The seating of the upper pod	12–15 cm	15–20 cm	14–18 cm
Time of maturity	Very early	Early	Medium
Vegetation period (in Poland)	100–130 days	125–135 days	135–140 days
Yield	4 t/ha	Over 4 t/ha	Over 4 t/ha
1,000 seeds weight	110–155 g	180 g	180–185 g
Protein content	36–40%	36–40%	40–44%
Fat content	17.5–21%	18–22%	17.4–19%

**Table 2:** Plant developmental stages in which the biostimulant was applied.

**Biostimulant: fylloton**			
**Single spraying Bbch 13–15**		**Double spraying Bbch 13–15, bbch 61**	
**Concentration (%)**	**Volume of working solution (L ha**^−1^**)**	**Concentration (%)**	**Volume of working solution (L ha**^−1^**)**
0.7	300	0.7	300
1	300	1	300
0.7	300	0.7	300
1	300	1	300

The biostimulant was applied at the BBCH 13-15 stage of soybean plants development, when foliar administration of microelement preparations is recommended to stimulate plant growth and development, especially after the occurrence of stress factors like low temperatures. The second spraying with the biostimulant was performed at the BBCH 62 stage of soybean plants development, i.e., at the beginning of blooming, because this is a critical period in their development when the optimal temperature should range from 15 to 19°C.

Due to the lack of any recommendations as for Fylloton doses applied in soybean cultivation, its two maximal doses recommended for other crops were used in this study.

Biostimulant was applied in the following periods in successive study years, depending on the developmental stage of the plants: 2014—single spraying on June 21 and double spraying on June 21 and July 5; 2015—single spraying on June 20 and double spraying on June 20 and July 3; 2016—single spraying on June 7 and double spraying on June 7 and June 23. Plants were sprayed with a biostimulant solution using a GARLAND FUM 12 B battery field sprayer at a constant pressure of 0.30 MPa, using 300 liters of solution per hectare.

The average temperature and rainfalls in the soybean growing season are shown in Table 3.

**Table 3:** Temperature (T) and rainfalls during the soybean growing season 2014–2016.

**Month**	**Year**						**Average from 2002 to 2013**	
	**2014**		**2015**		**2016**			
	**T (°C) (min/max)**	**Rainfall (mm)**	**T (°C) (min/max)**	**Rainfall (mm)**	**T (°C) (min/max)**	**Rainfall (mm)**	**T (°C)**	**Rainfall (mm)**
April	9.4 (−6.0/22.7)	36.5	8.2 (−1.7/24.3)	30.1	9.2 (−1.2/22.6)	68.4	8.5	41.2
May	13.7 (0.5/27.7)	208.3	12.7 (1.5/24.9)	108.6	13.8 (2.6/26.7)	61.3	12.7	63.4
June	16.1 (6.7/28.9)	67.1	17.4 (6.6/30.5)	14.1	18.1 (4.2/31.5)	97.1	17.7	68.6
July	20.3 (10.0/31.0)	104.2	19.6 (8.4/33.4)	59.2	19.5 (8.8/31.2)	107.6	18.9	79.1
August	18.2 (6.3/34.0)	115.4	21.6 (5.6/35.5)	23.4	18.2 (7.1/30.7)	95.3	19.4	71.8
September	13.7 (3.7/25.8)	89.4	15.1 (4.2/34.5)	137.6	15.2 (1.6/28.7)	41.2	14.1	69.2
Average/Total	15.1	620.9	15.8	373.0	17.1	470.9	15.2	393.3

### Plant yielding and nutritional value determination

The number of seeds per 1 m^2^, seed yield, thousand seed weight, number of pods per plant, location height of the first pod, number of nodes in the main shoot, height of plants, and protein and fat contents in seeds were determined.

Fat content was determined according to the acid hydrolysis method (AOAC, 2000, Official Method 922.86).

Protein content was determined by the Kjeldahl method (AOAC, 2000, Official Method 992.23, 979.09).

### Nutraceutical potential

#### Phenolics content and antioxidant capacity determination

Extract preparation. Soya flours (0.25 g in triplicate) were extracted three times with 4 mL of C3H6O/H_2_O/HCl (70:29:1, v/v/v) (Swieca et al., 2012). After centrifugation (6,800 × g, 10 min), the supernatant was collected and used for further analysis.

#### Phenolics determination

##### Determination of total phenolic compounds (TPC)

Determination of total phenolic compounds (TPC). The TPC was determined using the Folin-Ciocalteau reagent according to the method presented by Singleton and Rossi (1965). To 0.5 mL of the sample, 0.5 mL of distilled water and 2 mL of Folin-Ciocalteau reagent. After 3 min, 10 mL of 10% Na_2_CO_3_ were added and the contents were mixed and stand for half-hour. Absorbance was measured at 725 nm using a UV–vis spectrophotometer. The TPC was calculated as a gallic acid equivalent (GAE) in mg per g of dry matter (DM).

##### Determination of flavonoid content (TFC)

Determination of flavonoid content (TFC). The TFC was determined according to the method given by Lamaison and Carnet (1990). The 1 mL of the supernatant was mixed with 1 mL of a 2% AlCl3 × 6H_2_O solution (in methanol) and incubated at 20°C for 10 min. After incubation the absorbance was measured at 430 nm. TFC was calculated as a quercetin equivalent (QE) in mg per g of dry matter (DM).

##### Determination of anthocyanins (TAC)

TAC was evaluated according to the method based on absorbance measurements at different pH (Fuleki and Francis, 1968). Samples were combined (1:20, v:v) with potassium chloride and with sodium acetate buffers at pH 1.0 and 4.5. After 15 min of an equilibration period, the absorbance was measured at 520 and 700 nm. “A corrected absorbance value was calculated as [(A520–A700) pH 1.0 – (A520–A700) pH 4.5]. The anthocyanin content was calculated using the molar absorptivity (Є) and molecular weights (MW) of cyanidin 3-glucoside (Є = 26,900; MW = 449.2). Results were expressed as a cyanidin 3-glucoside equivalent (Cy3-GE) in mg per g of dry matter (DM)” (Kocira, A. et al., 2017).

#### Antioxidant activities

“Reducing power (RP). Reducing power was determined with the method of Pulido et al. (2000). The sample (2.5 mL) to be analyzed was mixed with phosphate buffer (2.5 mL, 200 mM, pH 6.6) and potassium ferricyanide K_3_[Fe(CN_6_)] (2.5 mL, 1%)” (Kocira, A. et al., 2017). “The mixture was incubated at 50°C for 20 min. Reactions were stopped with 0.5 mL of 10% TCA and centrifuged for 10 min (6,800 × g). The upper layer of the solution (2.5 mL) was mixed with distilled water (2.5 mL) and 0.5 mL of 0.1% FeCl_3_ and the absorbance was measured at 700 nm. Increased absorbance of the reaction mixture indicated an increase in reducing power. Reducing power was expressed as a Trolox equivalent in mg per g of dry matter (DM)” (Swieca et al., 2014).

### Statistical analysis

The obtained results were statistically elaborated with Statistica 13 software (StatSoft, Inc.). The materials were collected over three seasons (2014–2016). All nutritional and biological assays were performed in triplicate (unless otherwise stated). Normality of distribution of data was assessed by the Shapiro–Wilk test. The results were analyzed statistically using the one-way analysis of variance, ANOVA. Analyses were conducted for the effect of growing period, cultivar and use of the biostimulant on biometric traits, yield as well as nutritive and antioxidative properties of soybean seeds. The effect of the cultivar was depicted based on the difference between the mean result obtained after biostimulant application (AFT) and control (C). In statistical analyses, the number of replications for each combination (application) in each study year was *N* = 12. The number of replications for the application in the study period (2014-2016) was *N* = 36, whereas in a given season it was *N* = 60. The significance of differences between evaluated mean values was estimated by means of Tukey test intervals of confidence at a significance level of *p* < 0.05.

## Results

### Effect of fylloton on biometric characteristics of soybean plants

The best results in increasing the height of plants in Annushka cv. were achieved after double application of biostimulant at both concentrations (an increase by 19–21% in the average from study years compared to the control) (Table 4). Double plant spraying with Fylloton yielded good results in 2014 at the higher concentration of the biostimulant and in 2016 at its lower concentration. In addition, in the first and second year of the study, the single spraying of plants with Fylloton in the lower concentration stimulated plant growth. In turn, growth of plants of Mavka cv. was stimulated after double plant spraying with the higher concentration of Fylloton, which was confirmed by results obtained in individual study years. Considering both particular years and average of study years, Atlanta cv. responded positively to biostimulant application, because its plants grown in combinations with Fylloton were higher by 27–35% than plants from the control plot. The positive effect of biostimulant use on the number of nods in Annushka cv. plants was observed in 2014 after single spraying with both studied concentrations of Fylloton and in 2015 after double spraying with the lower concentration of the biostimulant. In Mavka cv., node number in the main shoot increased both after double spraying with the lower concentration in the case of average from years 2014 to 2016 and in the years 2014 and 2015, as well as after single spraying with the higher dose of the biostimulant in the case of average from study years and in the years 2014 and 2016. In turn, in Atlanta cv., the value of this trait was higher by 32–48% (for an average from 3 years) in all combinations with the biostimulant compared to the control plot in the case of the average from study years and for the year 2016. In plants of Annushka cv., the height of the first pod location on the shoot was higher in 2014 upon both single and double spraying with the higher concentration of Fylloton, whereas in 2016—after single spraying with the higher concentration. Mavka cv. responded with an increased height of the first pod in all combinations with biostimulant application in 2015. In turn, plants of Atlanta cv. were characterized by the highest height of pod location after single spraying with the lower dose of Fylloton in 2015. Such a correlation was demonstrated in all study years for Annushka cv. and Atlanta cv. as well as in 2015 and 2016 for Mavka cv. Annushka cv. responded positively to biostimulant application also in other combinations in 2014. In all soybean cultivars, the number of pods per plant (for an average from 3 years) was significantly higher than in the control after single treatment with the lower biostimulant concentration (47% increase for Annushka, 34% for Mavka and 43% for Atlanta). In addition, pod number was increased as a result of double plant spraying with the lower dose of the biostimulant in 2014 in Mavka cv. and as a result of double spraying with its higher dose in 2015 in the case of Atlanta cv.

**Table 4:** Effect of Fylloton treatment on biometric characteristics of soybean.

**Parameters**	**Fylloton treatment**	**Soya cultivar**											
		**Annushka**				**Mavka**				**Atlanta**			
		**Season**			**AA**	**Season**			**AA**	**Season**			**AA**
		**2014**	**2015**	**2016**		**2014**	**2015**	**2016**		**2014**	**2015**	**2016**	
Plant height [cm]	C	63.6^a^	48.1^a^	87.9^ab^	66.5^a^	82.5^a^	72.4^a^	80.4^a^	78.4^a^	85.1^a^	81.5^a^	88.8^a^	85.1^a^
	SS 0.7%	70.3^c^	70.7^c^	74.3^a^	71.8^ab^	99.7^bc^	85.8^b^	103.8^bc^	96.5^bc^	114.2^b^	102.3^b^	119.6^b^	112.0^b^
	DS 0.7%	76.4^b^	66.6^bc^	97.7^b^	80.2^b^	98.2^b^	79.7^ab^	86.3^a^	88.1^b^	121.5^b^	100.0^b^	122.8^b^	114.8^b^
	SS 1.0%	75.2^b^	62.5^b^	83.9^ab^	73.9^ab^	97.2^b^	85.8^b^	90.6^ab^	91.2^b^	124.1^b^	97.0^b^	121.6^b^	114.2^b^
	DS 1.0%	79.9^c^	67.4b^c^	89.8^ab^	79.0^b^	102.6^c^	100.3^c^	105.3^c^	102.7^c^	114.5^b^	95.5^b^	113.8^b^	107.9^b^
	AS	73.1^b^	63.1^a^	86.7^c^		96.0^b^	84.8^a^	93.3^b^		111.9^b^	95.3^a^	113.3^b^	
	AFT—C	9.7^a^				16.2^a^				27.1^a^			
	Season					2014: 93.7^ab^	2015: 81.0^a^	2016: 99.9^b^					
Number of nodes in the main shoot	C	11.3^b^	8.8^a^	14.2^a^	11.4^a^	8.8a	11.0^a^	10.5^a^	10.1^a^	11.2^a^	10.0^a^	9.6^a^	10.3^a^
	SS 0.7%	14.2^c^	11.7^bc^	18.9^a^	14.9^a^	10.5^b^	15.4^bc^	12.0^ab^	12.6^ab^	14.7^b^	15.3^a^	15.6^b^	15.2^b^
	DS 0.7%	8.8^a^	11.9^c^	20.5^a^	13.7^a^	12.6^c^	16.2^c^	13.0^ab^	13.9^b^	13.4^ab^	10.5^a^	16.7^b^	13.5^b^
	SS 1.0%	13.9^c^	8.2^a^	19.5^a^	13.9^a^	12.8^c^	10.2^a^	15.6^b^	12.9^b^	12.0^ab^	13.9^a^	14.9^b^	13.6^b^
	DS 1.0%	11.4^b^	9.4^ab^	16.2^a^	12.3^a^	12.0^c^	12.3^ab^	12.8^ab^	12.4^ab^	12.6^ab^	15.2^a^	16.0^b^	14.6^b^
	AS	11.9^a^	10.0^a^	17.9^b^		11.3^a^	13.0^b^	12.8^ab^		12.8^a^	13.0^a^	14.6^a^	
	AFT—C	2.3^a^				2.9^a^				4.0^a^			
	Season					2014: 12.0^a^	2015: 12.0^a^	2016: 13.5^a^					
Location height of the first pod [cm]	C	10.4^a^	5.8^a^	9.4^ab^	8.5^a^	14.1^a^	5.9^a^	13.1^a^	11.0^a^	16.2^a^	10.9^a^	14.1^a^	13.7^a^
	SS 0.7%	12.2^bc^	5.4^a^	7.4^a^	8.3^a^	12.9^a^	8.7^b^	18.1^a^	13.2^a^	11.9^a^	13.9^b^	11.4^a^	12.4^a^
	DS 0.7%	11.0^ab^	6.4^a^	8.0^ab^	8.4^a^	13.9^a^	10.1^b^	15.5^a^	13.2^a^	13.3^a^	13.6^ab^	12.3^a^	13.1^a^
	SS 1.0%	12.6^c^	6.0^a^	10.2^b^	9.6^a^	14.8^a^	10.4^b^	15.3^a^	13.5^a^	12.2^a^	12.6^ab^	12.8^a^	12.5^a^
	DS 1.0%	12.7^c^	5.8^a^	9.1^ab^	9.2^a^	14.5^a^	10.8^b^	13.5^a^	12.9^a^	14.3^a^	11.7^ab^	14.5^a^	13.5^a^
	AS	11.8^c^	5.9^a^	8.8^b^		14.0^b^	9.2^a^	15.1^b^		13.6^a^	12.5^a^	13.0^a^	
	AFT—C	0.4^a^				2.2^a^				−0.8^a^			
	Season					2014: 13.1^b^	2015: 9.2^a^	2016: 13.6^b^					
Number of pods [per plant]	C	17.3^a^	16.7^a^	19.8^a^	17.9^a^	14.8^a^	16.9^a^	15.5^a^	15.7^a^	15.2^a^	14.7^a^	16.3^a^	15.4^a^
	SS 0.7%	25.0^b^	26.4^c^	27.9^c^	26.4^c^	19.4^bc^	23.1^c^	20.7^b^	21.0^c^	21.5^b^	23.5^c^	21.0^c^	22.0^d^
	DS 0.7%	24.0^b^	19.7^ab^	22.0^ab^	21.9^b^	20.1^c^	18.2^ab^	19.0^ab^	19.1^b^	16.5^ab^	18.9^b^	18.2^b^	17.9^ab^
	SS 1.0%	22.0^b^	20.4^ab^	25.9^bc^	22.8^b^	19.0^b^	17.8^ab^	17.6^ab^	18.1^b^	19.6^ab^	17.6^b^	19.3^b^	18.8^bc^
	DS 1.0%	23.1^b^	22.1^b^	25.5^bc^	23.6^bc^	18.7^b^	19.6^b^	18.3^ab^	18.9^b^	17.2^ab^	24.9^c^	19.9b^c^	20.7^cd^
	AS	22.3^a^	21.0^a^	24.2^b^		18.4^a^	19.1^a^	18.2^a^		18.0^a^	19.9^c^	18.9^b^	
	AFT—C	5.8^a^				3.6^a^				4.5^a^			
	Season					2014: 19.5^a^	2015: 20.0^a^	2016: 19.0^a^					

*AS, Average season; AA, Average application; AFT, Average Fylloton treatment; C, control; SS 0.7%, single spraying by 0.7% solution of Fylloton; DS 0.7%, double spraying by 0.7% solution of Fylloton; SS 1%, single spraying by 1% solution of Fylloton; DS 1%, double spraying by 1% solution of Fylloton. Means in the columns, concerning the selected traits, followed by different small letters are significantly different at p < 0.05*.

### Effect of fylloton on soybean yield

Foliar application of Fylloton positively influenced the yield parameters of the three studied soybean cultivars (Table 5). The most promising results in increasing the number of seeds were found after double spraying of plants with 1% Fylloton solution, when this number was increased by 18% in Annushka cv., by 27% in Mavka cv. and by 23% in Atlanta cv. compared to the control (for an average from 3 years). A positive response of Atlanta cv. was also observed after double spraying with the lower concentration of Fylloton in the case of average from years 2014 to 2016 and in the years 2014 and 2015 (an increase in the value of this trait by 21% for year average compared to the control). In the first year of the study, a higher seed number was recorded in the other combinations with Fylloton use in the case of Mavka and Atlanta cultivars. In turn, Annushka cv. responded positively with an increased seed number after single spraying with the higher concentration of the biostimulant in 2014 and after single spraying with its lower concentration in 2016. The highest seed yield in the case of Mavka cv. and Atlanta cv., both considering average from study years and individual study years, was observed after double application of Fylloton in the higher concentration, when seed yield increased by 23 and 24% compared to the control (for an average from 3 years). Atlanta cv. achieved a high seed yield also in the other combinations with the biostimulant in 2014 and after double spraying with the lower concentration and after single spraying with the higher concentration of Fylloton in 2015. In turn, in the case of Annushka cv., the highest seed yield was obtained after single plant spraying with the lower concentration of the biostimulant for study year average (increase by 20%) and in 2016, although the double spraying with Fylloton in the higher concentration also ensured good seed yield seen from the perspective of study years average (increase by 18%) and in the years 2014 and 2015. The cultivar Annushka reacted by reducing one thousand seed weight after Fylloton application, especially after plants spraying with the higher biostimulant concentration (9–10% reduction for year average compared to the control). Only in the last year of the study, did the plants single-sprayed with the lower concentration of the biostimulant achieve thousand seed mass equal to that reported for control plants. No significant differences in the value of this trait, compared to control plants, were observed in the case of Mavka and Atlanta cultivars treated with Fylloton when analyzing the average of three study years. In turn, a higher thousand seed mass was demonstrated for Mavka cv. in 2014 after double spraying with the higher concentration of Fylloton, and also in 2015 and 2016 after single spraying with the lower dose as well as in 2016 after double spraying with the lower dose of the biostimulant. In the case of Atlanta cv., increased thousand seed mass was achieved in 2014 and 2015 after double plants spraying with the higher concentration of Fylloton and in 2016 after single spraying with Fylloton in the same concentration.

**Table 5:** Effect of Fylloton treatment on soybean seed yield.

**Parameters**	**Fylloton treatment**	**Soya cultivar**											
		**Annushka**				**Mavka**				**Atlanta**			
		**Season**			**AA**	**Season**			**AA**	**Season**			**AA**
		**2014**	**2015**	**2016**		**2014**	**2015**	**2016**		**2014**	**2015**	**2016**	
Number of seeds (per m^−2^)	C	2,142^a^	2,227^a^	2,210^a^	2,193^a^	2,091^a^	2,228^a^	2,199^a^	2,173^a^	1,993^a^	1,881^a^	2,007^a^	1,960^a^
	SS 0.7%	2,484^b^	2,714^bc^	2,959^b^	2,719^bc^	2,631^b^	2,352^a^	2,298^a^	2,427^b^	2,314^b^	2,228^b^	2,178^b^	2,240^b^
	DS 0.7%	2,374^b^	2,700^bc^	2,700^ab^	2,591^b^	2,665^b^	2,460^ab^	2,190^a^	2,438^b^	2,253^b^	2,551^d^	2,292^bc^	2,365^c^
	SS 1.0%	2,689^c^	2,604^b^	2,616^ab^	2,636^bc^	2,562^b^	2,626^b^	2,317^a^	2,502^b^	2,328^b^	2,404^c^	2,281b^c^	2,338^bc^
	DS 1.0%	2,781^c^	2,871^c^	2,819^b^	2,824^c^	2,666^b^	3,018^c^	2,589^b^	2,758^c^	2,313^b^	2,514^d^	2,384^c^	2,404^c^
	AS	2,494^a^	2,623^b^	2,661^b^		2,523^b^	2,537^b^	2,319^a^		2,240^a^	2,316^b^	2,228^a^	
	AFT—C	500^a^				359^a^				376^a^			
	Season					2014: 2419^a^	2015: 2492^a^	2016: 2381^a^					
Seed yield (t ha^−1^)	C	2.812^a^	2.859^a^	2.969^a^	2.880^a^	3.439^a^	3.215^a^	3.520^a^	3.391^a^	3.631^a^	3.168^a^	3.432^a^	3.410^a^
	SS 0.7%	3.073^b^	3.324^bc^	3.963^b^	3.453^b^	4.326^bc^	3.307^a^	3.762^a^	3.798^abc^	4.186^b^	3.567^b^	3.849^b^	3.867^b^
	DS 0.7%	3.078^b^	3.234^bc^	3.277^ab^	3.196^ab^	4.355^bc^	3.252^a^	3.583^a^	3.730^ab^	4.060^b^	4.082^c^	4.094^c^	4.079^bc^
	SS 1.0%	3.151^b^	3.152^b^	3.156^ab^	3.153^ab^	4.200^b^	3.812^b^	3.762^a^	3.925^bc^	4.065^b^	4.018^c^	4.111^cd^	4.065^bc^
	DS 1.0%	3.368^c^	3.446^c^	3.406^ab^	3.407^b^	4.497^c^	3.920^b^	4.137^b^	4.185^c^	4.282^b^	4.048^c^	4.333^d^	4.221^c^
	AS	3.096^a^	3.203^ab^	3.354^b^		4.163^c^	3.501^a^	3.753^b^		4.045^b^	3.777^a^	3.964^b^	
	AFT—C	0.422^a^				0.518^a^				0.648^a^			
	Season					2014: 3.768^ab^	2015: 3.494^a^	2016: 3.910^b^					
1,000 seed weight (g 1,000^−1^)	C	131.3^e^	128.4^b^	134.3^b^	131.3^c^	164.5^a^	144.3^bc^	160.1^a^	156.3^a^	182.2^ab^	168.5^b^	171.0^a^	173.9^a^
	SS 0.7%	123.8^c^	122.5^a^	133.9^b^	126.7^b^	164.4^a^	140.6^b^	163.7^b^	156.2^a^	180.9^ab^	160.1^a^	176.8^b^	172.6^a^
	DS 0.7%	129.3^d^	119.7^a^	121.4^a^	123.5^ab^	163.5^a^	132.1^a^	163.6^b^	153.1^a^	180.3^ab^	160.0^a^	178.6^bc^	173.0^a^
	SS 1.0%	117.2^a^	121.1^a^	120.7^a^	119.7^a^	164.0^a^	145.2^c^	160.9^a^	156.7^a^	174.7^a^	167.1^b^	180.2^c^	174.0^a^
	DS 1.0%	121.1^b^	120.0^a^	120.8^a^	120.6^a^	168.7^b^	129.8^a^	159.8^a^	152.8^a^	185.3^b^	161.1^a^	181.8^c^	176.0^a^
	AS	124.5^b^	122.3^a^	126.2^c^		165.0^c^	138.4^a^	161.6^b^		180.7^b^	163.4^a^	177.7^b^	
	AFT—C	−8.7^a^				−1.6^a^				0.0^a^			
	Season					2014: 156.7^ab^	2015: 141.4^a^	2016: 164.9^b^					

*Abbreviations: see Table 4. Means in the columns, concerning the selected traits, followed by different small letters are significantly different at p < 0.05*.

### Effect of fylloton on the nutritional properties of soybean seeds

**Figure 1:**
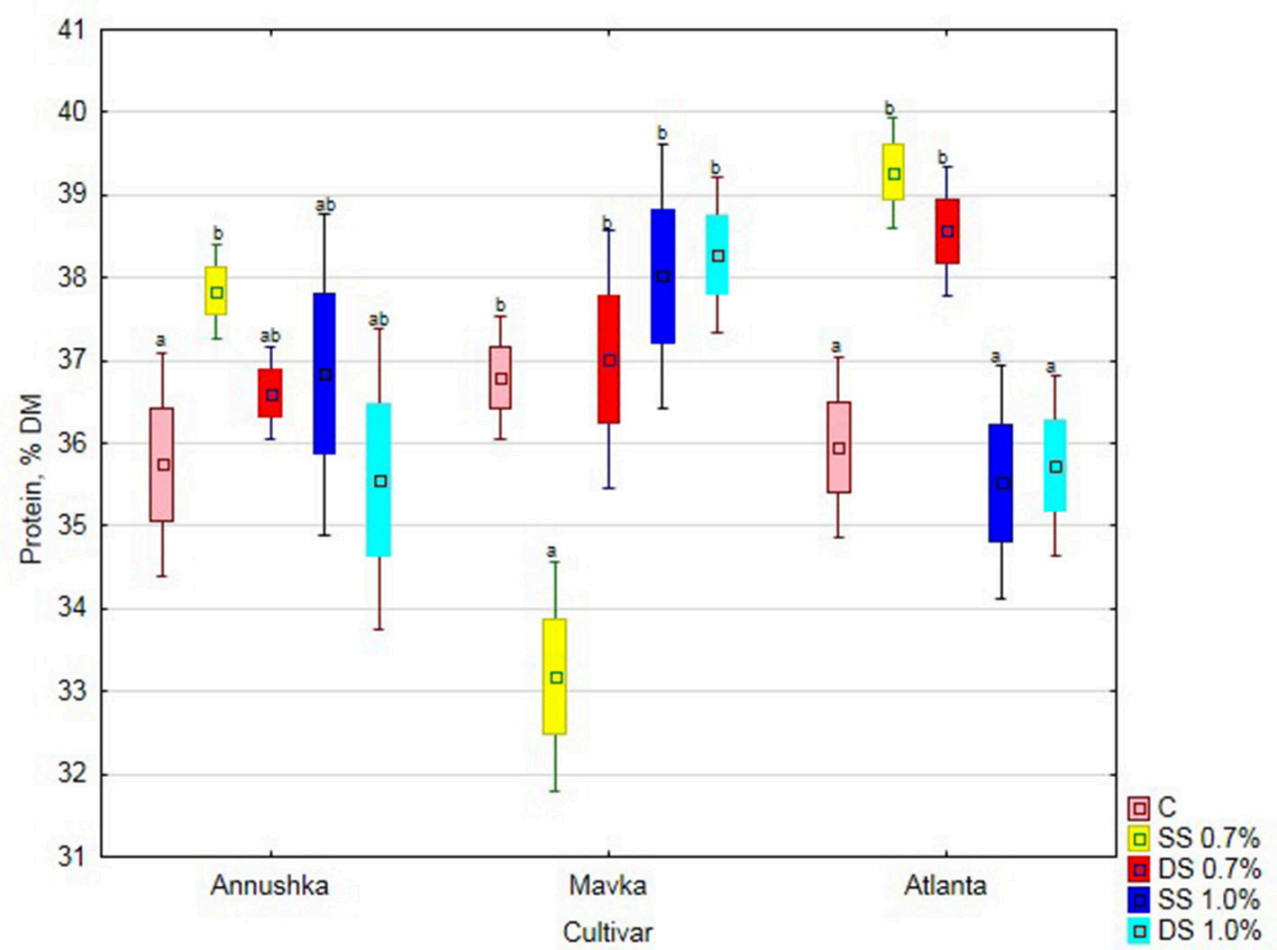
Effect of Fylloton treatment on protein content in three soybean cultivars—average from 2014 to 2016. Abbreviations: see Table 4.

The protein content of Annushka cv. soybean seeds significantly increased compared to the control after single application of the lower Fylloton concentration (6% increase). It was observed, however, that as the concentration and number of applications of this biostimulant increased, protein content of Annushka cv. seeds decreased (Figure 1). The cultivar Mavka responded with increased protein content as the concentration and number of biostimulant applications increased, reaching the highest value after double spraying with the higher concentration of the preparation (15% increase compared to single application of 0.7% Fylloton solution). The protein content of the control seeds was significantly different only upon the single spraying with the lower biostimulant concentration. The best results in increasing the protein content in Atlanta cv. soybean seeds were achieved after single treatment with 0.7% Fylloton, which yielded an increase of 9% compared to the control. The higher protein content was obtained at the lower concentration of the biostimulant, while its reduction was found at the higher concentration of the preparation, however the difference was not significant compared to the control.

**Figure 2:**
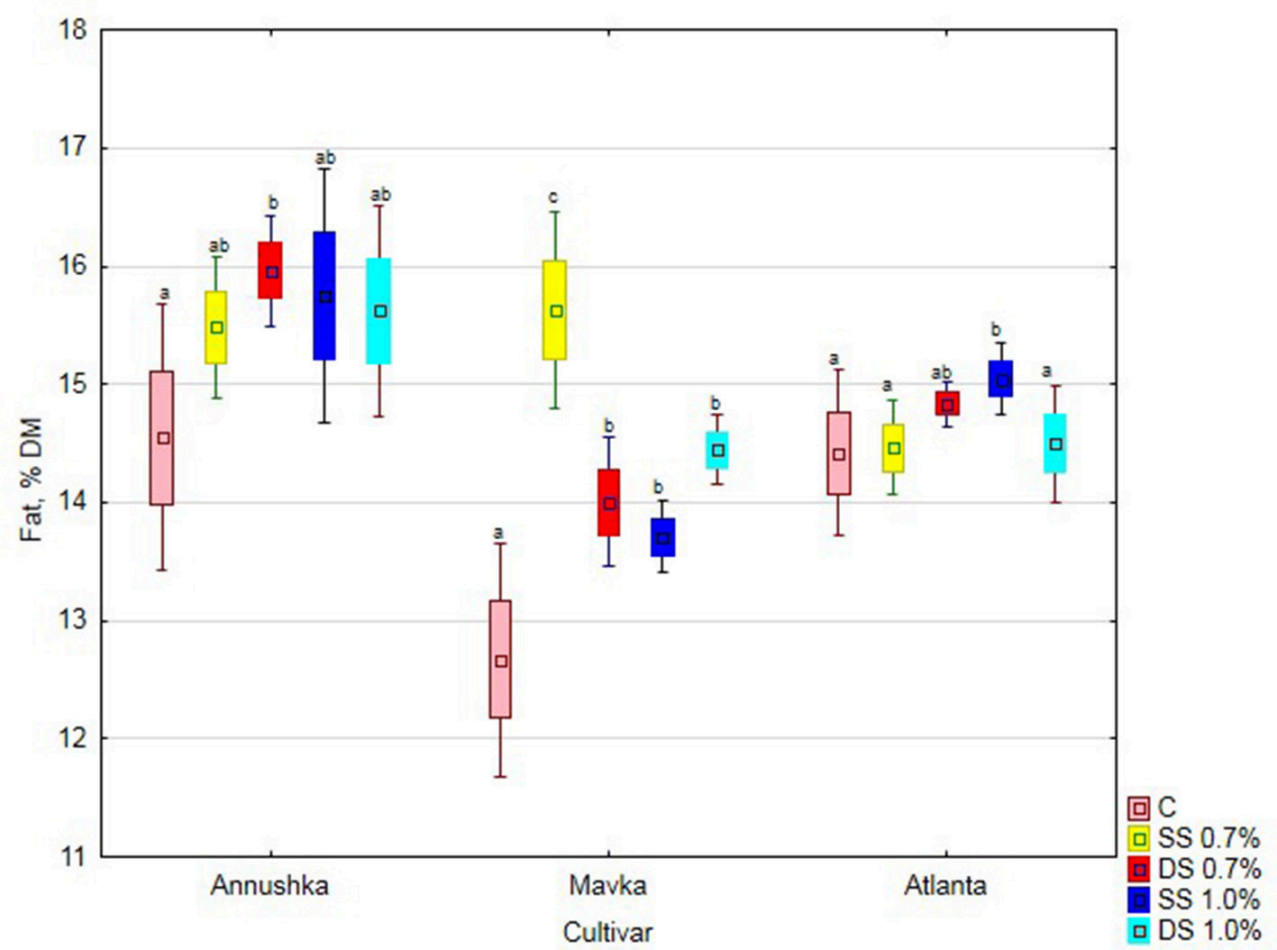
Effect of Fylloton treatment on fat content in three soybean cultivars—average from 2014 to 2016. Abbreviations: see Table 4.

There was a significant increase in fat content in Annushka a cv. soybean seeds both after single application of 1% Fylloton concentration and double application of 0.7% Fylloton concentration, i.e., by 8 and 10%, respectively, compared to the control (Figure 2). A clear increase in fat content in the seeds of Mavka cv. was obtained after single spraying of 0.7% biostimulant solution, i.e., by 23% compared to the control. In turn, the cultivar Atlanta responded positively to the single application of the higher biostimulant concentration, achieving a 4% increase in seed fat content compared to the control.

**Figure 3:**
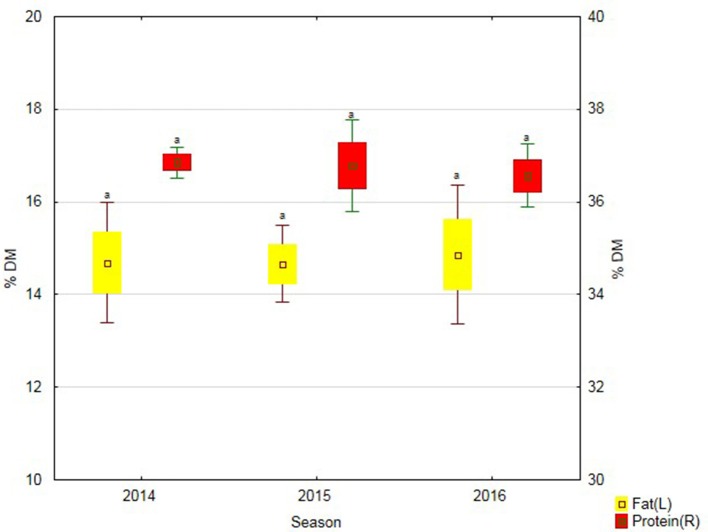
Protein and fat content in soybean seeds over three study years—average from soybean cultivars. Abbreviations: see Table 4.

Soybean treatment with the biostimulant contributed to an increased protein content in its seeds in 2015. In turn, in the last study year, the application of Fylloton caused the greatest increase in fats content in soybean seeds (Figure 3).

**Figure 4:**
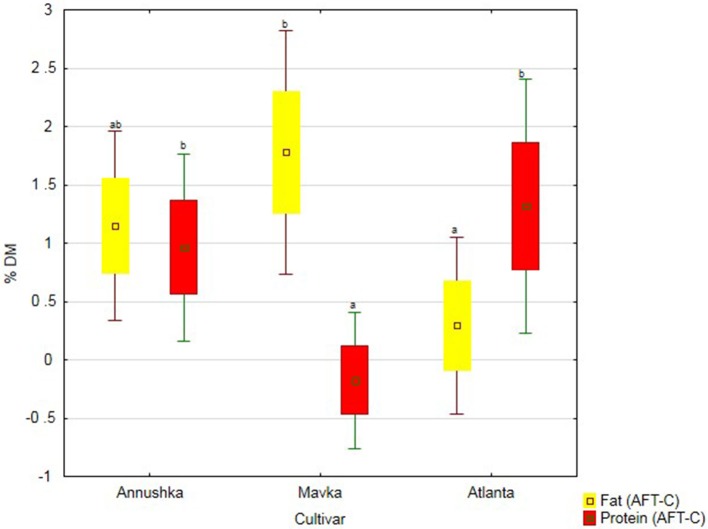
Protein and fat content in three soybean cultivars—average from 2014 to 2016. Abbreviations: see Table 4.

The greatest increase in fats content of seeds upon biostimulant application was observed for Mavka cv., whereas the greatest increase in protein content of the seeds—for Atlanta cv. (Figure 4).

### Effect of fylloton on the nutraceutical properties of soybean seeds

The use of Fylloton biostimulant affected polyphenolics content (TPC) in the seeds of the test cultivars (Table 6). Significant differences in TPC were observed for Annushka and Atlanta soybean cultivars. In both of these cultivars, the highest content of phenolic compounds was acquired after single treatment with 1% Fylloton solution, as the polyphenol content was nearly 2.5-fold higher in Atlanta cv. and 41% higher in Annushka cv. compared to the control. In relation to the control, an increasing tendency in contents of these bioactive compounds was observed only after spraying Mavka soybean seeds with the higher Fylloton concentration.

**Table 6:** Effect of Fyllloton treatment on phenolic contents and antioxidative activity of soybean seeds.

**Parameters**	**Fylloton treatment**	**Soya cultivar**											
		**Annushka**				**Mavka**				**Atlanta**			
		**Season**			**AA**	**Season**			**AA**	**Season**			**AA**
		**2014**	**2015**	**2016**		**2014**	**2015**	**2016**		**2014**	**2015**	**2016**	
Total phenols (mg g^−1^ DM)	C	4.12^b^	4.67^c^	4.40^c^	4.40^ab^	3.90^a^	4.82^a^	3.31a	4.01^a^	2.97^a^	3.05^a^	3.18^a^	3.06^a^
	SS 0.7%	4.36^c^	3.22^a^	3.56^b^	3.71^a^	4.59^ab^	5.98^d^	4.15^b^	4.91^a^	6.55^d^	6.42^d^	7.36^d^	6.78^cd^
	DS 0.7%	3.12^a^	3.95^b^	2.05^a^	3.04^a^	4.79^ab^	5.25^b^	4.65^d^	4.90^a^	6.21^c^	5.40^c^	4.65^c^	5.42^bc^
	SS 1.0%	7.26^e^	5.30^d^	6.09^e^	6.22^b^	5.46^b^	6.51^e^	4.36^c^	5.44^a^	8.24^e^	6.84^e^	7.60^e^	7.56^d^
	DS 1.0%	6.21^d^	5.36^d^	5.65^d^	5.74^b^	5.62^b^	5.87^c^	4.64^d^	5.38^a^	4.02^b^	4.15^b^	4.51^b^	4.23^ab^
	AS	5.01^c^	4.50^b^	4.35^a^		4.87^b^	5.69^c^	4.22^a^		5.60^a^	5.17^a^	5.46^a^	
	AFT—C	0.28^a^				1.15^a^				2.93^b^			
	Season					2014: 5.10^a^	2015: 5.20^a^	2016: 4.66^a^					
Total flavonoids (mg g^−1^ DM)	C	5.33^c^	4.43^c^	5.97^d^	5.24^b^	4.89^d^	3.45^c^	4.41^d^	4.25^b^	6.46^a^	5.91^a^	5.76^a^	6.04^a^
	SS 0.7%	5.70^d^	4.71^d^	5.20^c^	5.20^b^	2.33^b^	2.93^b^	2.41^c^	2.56^a^	11.74^e^	10.28^d^	11.92^e^	11.31^d^
	DS 0.7%	3.07^a^	2.24^a^	2.91^a^	2.74^a^	1.43^a^	1.69^a^	1.72^a^	1.61^a^	7.33^b^	5.95^a^	6.05^b^	6.44^ab^
	SS 1.0%	4.33^b^	2.96^b^	3.73^b^	3.67^ab^	2.65^c^	2.93^b^	2.24^b^	2.61^a^	7.99^c^	7.41^b^	7.76^c^	7.72^bc^
	DS 1.0%	6.20^e^	4.67^d^	5.20^c^	5.36^b^	5.02^e^	4.64^d^	4.56^e^	4.74^b^	8.20^d^	7.80^c^	8.11^d^	8.04^c^
	AS	4.93^c^	3.80^a^	4.60^b^		3.26^c^	3.13^b^	3.07^a^		8.34^c^	7.47^a^	7.92^b^	
	AFT—C	−1.00^a^				−1.37^a^				2.34^b^			
	Season					2014: 5.51^a^	2015: 4.80^a^	2016: 5.20^a^					
Anthocyanin (mg g^−1^ DM)	C	0.01^a^	0.01^a^	0.01^a^	0.01^a^	0.01^a^	0.02^a^	0.01^a^	0.01^a^	0.04^b^	0.05^b^	0.06^b^	0.05^b^
	SS 0.7%	ND	ND	ND	ND	0.01^a^	0.02^a^	0.01^a^	0.01^a^	0.02^a^	0.01^a^	0.01^a^	0.01^a^
	DS 0.7%	ND	ND	ND	ND	ND	ND	ND	ND	ND	ND	ND	ND
	SS 1.0%	0.01^a^	0.01^a^	0.01^a^	0.01^a^	ND	ND	ND	ND	ND	ND	ND	ND
	DS 1.0%	ND	ND	ND	ND	ND	ND	ND	ND	0.09^c^	0.10^c^	0.09^c^	0.09^c^
	AS	0.004^a^	0.004^a^	0.004^a^		0.004^a^	0.008^b^	0.004^a^		0.03^a^	0.03^a^	0.03^a^	
	AFT—C	−0.010^a^				−0.013^a^				−0.023^a^			
	Season					2014: 0.011^a^	2015: 0.013^a^	2016: 0.010^a^					
Reducing power (mg TE g^−1^dw)	C	0.31^ab^	0.26^bc^	0.33^b^	0.30^ab^	0.15^a^	0.18^a^	0.18^a^	0.17^a^	0.22^a^	0.25^ab^	0.26^b^	0.24^a^
	SS 0.7%	0.28^a^	0.19^a^	0.25^a^	0.24^a^	0.20^ab^	0.18^a^	0.17^a^	0.18^a^	0.29^b^	0.28^b^	0.27^b^	0.28^a^
	DS 0.7%	0.45^c^	0.35^d^	0.37^b^	0.39^b^	0.32^c^	0.29^c^	0.25^b^	0.29^b^	0.24^ab^	0.22^a^	0.27^b^	0.24^a^
	SS 1.0%	0.36^b^	0.25^b^	0.37^b^	0.33^ab^	0.29^cd^	0.21^ab^	0.20^ab^	0.23^ab^	0.24^ab^	0.28^b^	0.28^b^	0.27^a^
	DS 1.0%	0.35^b^	0.29^c^	0.32^ab^	0.32^ab^	0.24b^c^	0.25^bc^	0.21^ab^	0.23^ab^	0.22^a^	0.26^ab^	0.20^a^	0.23^a^
	AS	0.35^b^	0.27^a^	0.33^b^		0.24^b^	0.22^ab^	0.20^a^		0.24^a^	0.26^a^	0.26^a^	
	AFT—C	0.02^a^				0.06^a^				0.02^a^			
	Season					2014: 0.28^a^	2015: 0.25^a^	2016: 0.26^a^					

*Abbreviations: see Table 3. ND, not noted. Means in the columns, concerning the selected traits, followed by different small letters are significantly different at p < 0.05*.

The content of polyphenols in soybean seeds depended not only on the cultivar, but also on study year. In the first year of the experiment, the content of phenolic compounds was the highest compared to the other years in seeds of Annushka and Atlanta cultivars. The complex analysis of the average effect of meteorological conditions showed no significant differences in phenolics content between soybean seeds. The greatest difference in the content of polyphenols between the combinations treated with the biostimulant and the control (AFT-C) was found in the case of Atlanta cv. seeds.

There was a significant influence noted of biostimulant application when analyzing the content of flavonoids. The greatest variation was recorded for the cultivar Atlanta. The highest content of flavonoids in seeds of this cultivar was found after single application of biostimulant at a concentration of 0.7% (nearly 2-fold increase compared to the control) in relation to other cultivars analyzed. In turn, Annushka and Mavka cultivars responded by a decrease of flavonoid contents after Fylloton application. The content of the analyzed compounds was increased only after double treatment with biostimulant at its higher concentration, i.e., by 2 and 11%, respectively compared to the control, but these differences were statistically insignificant. A positive value of difference in flavonoids content between combinations treated with Fylloton and control samples (AFT-C = 2.34) was determined only for seeds of Atlanta cv. The content of flavonoids was the highest in seeds of all analyzed cultivars in 2014, compared to the two successive years of the study.

The presence of anthocyanins was detected only in 7 out of 15 analyzed biostimulant combinations in three soybean cultivars. In the case of Annushka cv., anthocyanins were identified in the control sample and after single treatment with the higher concentration of Fylloton, but no significant differences were found between these values. On the other hand, higher anthocyanin content was also recorded for the cultivar Mavka in the control sample and in seeds obtained from plants sprayed once with a 0.7% biostimulant solution. The increase of anthocyanin content was observed in Atlanta cv. after a double foliar application of a 1% Fylloton solution. The statistical analysis conducted for contents of anthocyanins showed that neither cultivar nor conditions occurring during plants growth had a significant effect on their content in soybean seeds.

The reducing power was determined to evaluate the effect of the foliar application of Fylloton on the antioxidative activity of the tested soybean cultivars. Almost all applied biostimulant combinations increased its value. Changes recorded in seeds of Annushka and Mavka cultivars were statistically significant (*p* < 0.05). The highest reducing power was found after double spraying with a 0.7% biostimulant solution, giving a 1.7-fold increase in Mavka cv. and a 27% increase in Annushka cv. compared to the controls, although the value obtained was not significantly different from the control. In seeds of Atlanta cv., there were no significant differences in the reducing power upon Fylloton application, but only an increasing tendency of this parameter after single spraying with the biostimulant at its both concentrations. A positive value of difference in reducing power between the combinations treated with Fylloton and the control samples (FT-C) was noted for all studied cultivars, however the highest value was computed for seeds of Mavka cv. The value of reducing power depended not only on the cultivar but also on study year. In 2014, its value was the highest in seeds of Annushka cv. and Mavka cv., compared to the other years of the study.

## Discussion

The increased awareness of agricultural producers as well as the possibility of using biostimulants in organic farming have contributed to the growing interest in the possibility of their application in plant cultivation. Biostimulants promote the growth of the aboveground and underground parts of plants, improve the uptake of water and nutrients, which results in increased biomass and crop yields. In addition, they improve crop quality, particularly when stressors occur. They stimulate the growth and development of plants, starting with the early stages of their development, as evidenced by numerous reports in the literature (Craigie, 2011; Calvo et al., 2014; Sharma et al., 2014; Bulgari et al., 2015; Colla et al., 2015; Yakhin et al., 2017). The response of plants to biostimulants is not always positive, and there are many studies that have not proven beneficial effects of their use, especially when grown under optimum conditions (Beckett and Van Staden, 1989; Aguirre et al., 2009; Kunicki et al., 2010).

The differences in plant responses to Fylloton may occur within the species, however, foliar application of this biostimulant has positively affected the growth of the aboveground part and the yield of all three soybean cultivars analyzed in our study. The double spraying of soybean with Fylloton stimulated plant growth, seed number and yield, with the strongest response observed in the case of Mavka cv. Accordingly to the phenotypic traits of this cultivar, its plants reach the height of 80-110 cm and yield above 4 t·ha^−1^. In our experiment, plants from control plots reached the minimal typical values of height and yield, whereas the use of the biostimulant increased values of these traits in treated plants. In turn, the single spraying of soybean with the lower concentration of Fylloton hat the most positive effect on pod number increase, especially in Annushka cv. The advisability of a few-fold application of the biostimulant was confirmed by Kowalska et al. (2012). Results of our experiment indicate that both the concentration and number of applications of the biostimulant have various effects on the morphological traits as well as on the size and quality of crop yield.

There have been no reports so far of the effects of biostimulants, containing both seaweed extract and amino acids, on plant growth. Therefore, the use of Fylloton biostimulant seems to be particularly beneficial, since the effects of preparations containing one of these components on the growth and development of crop plants have been well researched.

Biostimulants based on seaweed extracts stimulate the growth and development, which also has an impact on plant yields due to the content of macro- and micronutrients and various bioactive compounds, such as phytohormones. Seaweed contain cytokines, such as zeatin, isopentanyldeosine and small quantities of dihydrozeatine, betaine, ortho-topoline, and meto-topoline (Stirk et al., 2003, 2009; Yokoya et al., 2010). Among auxins, IAA (indolyl-3-acetic acid), ICA (indolyl-3-carboxylic acid), and IAM (indolyl-3-acetamide) are most commonly found in seaweed extracts (Yokoya et al., 2010).

Numerous studies confirmed the positive effect of seaweed extracts on the growth of *Fabaceae* and other crops. The application of seaweed extracts was proved to increase the height of soybean plants (15% *Kappaphycus alvarezii* extract) (Rathore et al., 2009), mung bean (1% *Sargassum wightii* extract and 3% *Caulerpa racemosa* extract) (Kumar et al., 2012; Sujatha and Vijayalakshmi, 2013), common bean (25% extract from *Fucus spiralis* or *Ulva rigida*) (Latique et al., 2013), and chickpea (1% *Ascophyllum nodosum* extract) (Boghdady et al., 2016). However, Kumar et al. (2012) have shown that increasing the *Sargassum wightii* extract to 2% resulted in a significant reduction in bean plant height. Foliar application of seaweed extracts (*Corallina elongate, Sargassum latifolium*) also increased the height of other crops, for example wheat (Ismail, 2016), eggplant (Abd El-Gawad and Osman, 2014), tomato (Sutharsan et al., 2014), and onion (Shafeek et al., 2015). In turn, the use of high concentrations of *Sargassum crassifolium* extract (100%) has been shown to inhibit the growth of tomato plants (Sutharsan et al., 2014). Marhoon and Abbas (2015) demonstrated that the response of plants to the seaweed extract was dependent on the cultivar, as it increased the height of paprika plants of California Wonder cv. by 70% and only by 40% in the case of Flavio F1 cv. Additionally, El-Miniawy et al. (2014) confirmed that the number of applications significantly influenced the growth of the plants, resulting in an increase in strawberry height of 21–30% after three applications of Algreen (extracts from *Sargassum* sp., *Ascophyllum nodosum, Laminaria* sp.) at the higher concentration (2 mL L^−1^).

A positive effect of seaweed extracts on crop yields has been confirmed by many authors. Legumes have been shown to favorably respond to foliar application of seaweed extract (*Caulerpa racemosa, Ascophyllum nodosum, Ecklonia maxima, Sargassum wightii, Kappaphycus alvarezii*) by increasing the number of pods and seeds, one thousand seed weight and seed yield in soybean (Rathore et al., 2009; Karthikeyan and Shanmugam, 2016), common bean (Zewail, 2014; Abo-Sedera et al., 2016; Kocira et al., 2016; Kocira, S. et al., 2017), mung bean (Kumar et al., 2012; Sujatha and Vijayalakshmi, 2013), chickpea (Boghdady et al., 2016), and fava bean (Jasim and Obaid, 2014); however, the traits studied were dependent on the cultivar and genus, as well as concentration and number of seaweed extract applications. Nevertheless, some authors have demonstrated that seaweed extracts do not affect or negatively affect plant yields, as compared to control, such as foliar application of higher concentrations of *Sargassum wightii, Sargassum crassifolium, Kappaphycus alvarezii*, and *Ascophyllum nodosum* that reduced the number of pods of mung bean (Kumar et al., 2012) and fruit yield of tomato (Mikiciuk and Dobromilska, 2014) and okra (Zodape et al., 2008).

Our earlier researches demonstrated that the size and quality of common bean yield depended on both concentration and number of applications of an extract from *Ecklonia maxima* (Kelpak SL) as well as on bean cultivar. The single spraying with the higher concentration of the biostimulant increased seed number and mass and pod number in Aura cv., whereas the use of the lower concentration of the biostimulant at the same developmental stage of plants increased seed number and mass in Toska cv. (Kocira et al., 2016). The double application of the same biostimulant increased rapeseed yield (Matysiak et al., 2012a). In addition, Dobromilska et al. (2008) and El-Miniawy et al. (2014) achieved a significant increase in tomato and strawberry yields after 3-fold foliar application of an extract from *A. nodosum*.

Similarly as phytohormones, the use of a protein hydrolysate containing plant-derived amino acids and peptides has been shown to induce plant growth and nitrogen uptake, contributing to an increased plant yield (Colla et al., 2014).

In turn, the positive effect of biostimulants based on amino acids on the growth, development, and yielding of plants is probably due to the fact that they stimulate the plant's defensive response to biotic and abiotic stressors at the molecular level (Cambri et al., 2008). In addition, amino acids contained in them are easily absorbed by plants, participate in the synthesis of many organic compounds, and also affect the uptake of macro- and micronutrients (Maini, 2000). Garcia et al. (2011) demonstrated that foliar application of amino acids and peptides together with nutrients increased the levels of potassium, calcium, magnesium, iron, copper, and zinc in leaves, affecting the nutritional status of leaves, which promoted better growth and development of plants. In addition, foliar applications of these preparations showed a phytohormone-like effect (Colla et al., 2014).

Colla et al. (2014) confirmed that the use of Trainer protein hydrolysate stimulated elongated growth of pea shoot, increasing the height of dwarf plants by 33%, i.e., acting similarly to gibberellin. In addition, foliar application of amino acids increased the height of legumes, for example, fava bean (El-Ghamry et al., 2009; Sadak et al., 2015), common bean (Abdel-Mawgoud et al., 2011; Zewail, 2014), and mung bean (Khalilzadeh et al., 2012), especially when grown under stressful conditions. In addition, Colla et al. (2014) have also demonstrated the stimulatory effect of a protein hydrolysate on the growth of the aboveground and underground parts of tomato seedlings, similar to that of auxin effect. El-Gamal et al. (2016) demonstrated that foliar application of amino acids positively influenced plant growth and development by increasing the content of phytohormones, e.g., by causing a nearly 2.5-fold increase in contents of gibberellin and cytokinin, and a 9-fold increase in auxin content in sugar beet shoots. There are also reports of slight effects of amino acids on plant height (increase by 1–7%), e.g., onion (Kandil et al., 2013), winter rapeseed (Wójtowicz, 2012), fennel (El-Bassiony et al., 2014), and wheat (Azimi et al., 2013), also grown under stressful conditions.

In an earlier study, common bean plants responded positively to the foliar application of Terra Sorb Complex (free amino acids), but the yield effect was dependent on the cultivar, concentration and number of biostimulant applications and climatic conditions in a given year A significant increase in seed number and mass and in pod number was observed after single spraying of bean with a lower concentration of this biostimulant in the case of Toska cv., and with its higher concentration in the case of Aura cv. (Kocira et al., 2015b). Foliar use of biostimulants containing amino acids positively influenced the yield of other legumes by increasing the number of seeds, pods, 100 seed weight and seed yield of fava bean (El-Ghamry et al., 2009; Sadak et al., 2015), pea (Shafeek et al., 2014), and common bean (Abdel-Mawgoud et al., 2011; Zewail, 2014). Some authors have demonstrated that the foliar application of amino acids resulted in nearly 2.5-fold increase in the number of fava bean pods (El-Ghamry et al., 2009) and pea seed yield (Shafeek et al., 2014). Jakiene (2013) and Wójtowicz (2012) confirmed the stimulating effect of the Terra Sorb Foliar biostimulant on rapeseed yielding, however, the yield was dependent on the application date and the plant developmental stage. There are also reports on the lack of influence of preparations containing amino acids on plant yield, for example, no effect of Aminoplant was found on the yield of *Cichorium endivia* (Gajc-Wolska et al., 2012) and spinach (Kunicki et al., 2010). However, the negative effect of amino acids: glutathione, cysteine and methionine (50 g L-1), on onion yield has been proved as well (El Awadi and Abd El Wahed, 2012).

There are studies on the effect of seaweed extract and amino acid applications on nutritive value and nutraceutical quality of plant products (Pise and Sabale, 2010; Fan et al., 2013; Kocira et al., 2015b, 2016; Kocira, A. et al., 2017; Kocira, S. et al., 2017; Zarzecka and Gugała, 2018), but there are no reports demonstrating such plant responses to the combined use of these components. In the current study, foliar application of Fylloton increased protein and fat contents in soybean seeds, although differences in the content of these compounds were dependent on the cultivar, number of applications and biostimulant concentration.

Seeds of Mavka cv. have the highest content of fats among all studied cultivars and the value of this trait was additionally increased as a result of biostimulant application. In turn, seeds of Atlanta cv. are characterized by the highest protein content, which was also significantly increased after plants treatment with the biostimulant. Both the number of biostimulant applications and its concentration affected yield quality, and the differences obtained in the study were often dependent on the cultivar. Increased contents of protein in seeds of Annushka and Atlanta cvs. and of fat content in seeds of Mavka cv. were determined after single spraying with the biostimulant in the lower concentration. In the case of Annushka cv., fat content of its seeds was also increased after double application of the same concentration of Fylloton. In turn, the highest fat content of seeds was achieved after single spraying the plants with the higher concentration of the biostimulant in the case of Annushka and Atlanta cultivars, and that of protein—after double plant spraying with the same concentration of Fylloton in the case of Mavka cv.

Matysiak et al. (2012b) demonstrated that protein content in wheat grain depended not only on the number of applications of an algae-based biostimulant (Kelpak SL), but also on the developmental stage of plants. The single or double application of the biostimulant in the later developmental stages of wheat had a positive effect on protein content in its seeds.

Jasim and Obaid (2014), Zewail (2014), and Kocira, S. et al. (2017) found that the foliar application of a seaweed extract had a positive effect on protein content in fava bean and bean seeds. Treatment of wheat plants with *Ulva rigida* and *Sargassum latifolium* extract resulted in increased protein content in grains (Ismail, 2016). However, other studies have shown that biostimulants do not always exert a positive effect on this trait. Foliar application of *Ecklonia maxima* extract reduced the protein content (albumin + globulin) in white-seeded bean seeds (Kocira et al., 2016).

The application of biostimulant containing free amino acids increased protein content in bean seeds (Zewail, 2014; Kocira et al., 2015b), pea (Shafeek et al., 2014), and fava bean (Sadak et al., 2015). Winter rapeseed spraying with Terra Sorb Foliar containing free amino acids slightly increased the content of fat and protein in seeds (Jakiene, 2013), although there were also reports which showed no effect of this biostimulant on fat content in these plants (Wójtowicz, 2012).

Our earlier researches confirmed plant response to be affected by the number of applications of a biostimulant based on amino acids. The highest content of protein in bean seeds was determined after single application of Terra Sorb Complex in the lower concentration in the case of Toska cv., and after single application of this biostimulant in the higher concentration in Aura cv. (Kocira et al., 2015b).

Our experiments demonstrated differences in the content of phenolic compounds, flavonoids, and anthocyanins in soybean seeds. The observed changes in Fylloton-treated crops were statistically different for most of the analyzed seeds when compared to the control seeds. It can be hypothesized that these changes could be related to the cross reaction between abiotic and biotic factors that induce stress in plants. Stress can affect plant metabolism, which in turn leads to enhanced antioxidative properties (Tenhaken, 2015; Złotek et al., 2016). In addition, seaweed extracts have also been shown to affect plant metabolism (Khan et al., 2009), and recent gene expression analyses have provided preliminary insights into some metabolic pathways. Fan et al. (2013) observed an increase in total protein content, antioxidative capacity, and phenolic and flavonoid contents in spinach treated with *Ascophyllum nodosum* extract. This was due to the increase in the number of transcripts of key enzymes involved in nitrogen metabolism (cytotoxic glutamine synthetase), antioxidative capacity (glutathione reductase), and glycine betaine synthesis (betaine dehydrogenase and choline monohydrate).

The nutraceutical potential of plant products is primarily related to the presence and content of antioxidants (Scalbert and Williamson, 2000). In the present study, the total polyphenolics content was increased in soybean seeds of Annushka, Atlanta and Mavka cultivars as a result of Fylloton biostimulant treatment. Similar conclusions were drawn by Pise and Sabale (2010), who also noted an increase in polyphenol levels after treatment of fenugreek plants with seaweed extract (*Ulva fasciata, Sargassum illicifolium, Gracilaria corticata*). Previous studies conducted by Kocira, A. et al. (2015, 2017), and Kocira et al. (2016) also demonstrated that the use of Nano-Gro, Kelpak (*Ecklonia maxima* extract), and Atonik biostimulants increased both the content of polyphenolic compounds and the antioxidative potential of common bean. Our experiments and other studies have shown that both contents of bioactive compounds and antioxidative potential depend on the types of biostimulants used, their concentrations and the number of applications and the subsequent treatment of the raw material (Kocira, A. et al., 2015, 2017; Oniszczuk et al., 2015; Bouasla et al., 2016; Kocira et al., 2016; Nadulski et al., 2016).

The reducing power, expressed as Trolox equivalent, did not differ significantly between the seeds obtained from Fylloton-treated plants and controls. However, an increase in the reducing power upon plant spraying with 1% biostimulant concentration, both in single and double spraying configurations. Similar observations have been made by Koleška et al. (2017), who evaluated the effect of Viva biostimulant (containing, among others, amino acids) and different fertilization on the antioxidative activity and phenolics content in tomato plants. These authors found that the increase in polyphenolics content under conditions of reduced nitrogen fertilization could be due to the increased alanine ammonia-lyase (PAL) activity, which resulted in the production of cinnamic acid needed for the biosynthesis of flavonoids and amino acid groups (Kovačik and Bačkor, 2007). Antioxidative activity often increases in many plants, because of the increased polyphenolics content, as phenolic functional groups serve as free radical sequestrants (Pantelidis et al., 2007; Du et al., 2009). On the other hand, the use of different biostimulants can significantly affect the phenylpropanoid pathway. In this case, contents of different classes of phenolic compounds, especially gallic acid, flavanones and stilbenes, were observe to increase (Pardo-Garcia et al., 2014).

Results of our study and findings of other authors prove that both contents of bioactive compounds and antioxidative potential depend on the type of biostimulants, their concentrations and number of their applications. It was also found that contents of polyphenols, flavonoids and anthocyanins, and reducing power of soybean seeds were affected not only by soybean cultivar but also by study year. Differences were observed between contents of bioactive compounds in seeds from 2014 and from other years of the experiment. They could be due to conditions occurring in the last 2 months of the growing period of soybean, when a decrease was noted in the average air temperature and an increase in sum of precipitation compared to average values from the multi-year period. It additionally resulted in extended period of seed ripening. Grabowska et al. (2012) also reported that carrot response to the use of a biostimulant based on amino acids depended mainly on the analyzed cultivar and conditions during growing season. Temperature and atmospheric precipitation during soybean vegetation in 2014 could be stress factors to its plants. This is confirmed by results obtained by Ertani et al. (2013) who reported increased contents of phenols and flavonoids in corn plants grown under stress conditions. It proves these compounds to be important antioxidants that may contribute to plant response to stress conditions. The increase in flavonoids content is also associated with variability in the activity of the key enzyme (PAL) engaged in the biosynthesis of phenylpropanoids. It was reported that the gene encoding this protein responds to many abiotic and biotic stress factors in many plant species and may be induced by biostimulants (Schiavon et al., 2010; Ertani et al., 2011).

The concentration of a biostimulant and number of its applications as well as plant cultivar and atmospheric conditions may affect biostimulant action and also plant response to stress factors manifested in changing contents of antioxidants.

It was demonstrated that when coupled with amino acids the extracts from seaweeds, rich in polysaccharides, microelements and plant growth hormones, had a positive effect on plant growth and increased plant resistance to both abiotic and biotic stress factors (Khan et al., 2009; Craigie, 2011; González et al., 2013). It needs to be emphasized, however, that mechanisms of their action are not well understood and elucidated yet, but the use of cutting-edge analytical and molecular tools may bring a new insight into their effects on gene expression, biochemical pathways, and physiological processes (Rayirath et al., 2009; Nair et al., 2012; Jannin et al., 2013; Wally et al., 2013). Wider and deeper understanding of the mechanisms of action of biostimulants based on extracts from seaweeds and amino acids may be helpful in optimizing their use in sustainable management of agricultural systems (Khan et al., 2009; Quilty and Cattle, 2011).

## Conclusions

In conclusion, the cultivar, number of treatments and concentration of Fylloton biostimulant strongly determined the final results. Based on the conducted experiments, it can be concluded that Fylloton treatment is an effective way to improve soybean growth and yield without negatively affecting the nutraceutical and nutritional quality of its seeds. Double spraying with the higher Fylloton concentration stimulated plant growth and increased seed number and yield. In contrast, single treatment with the lower biostimulant concentration beneficially affected the number of pods. A positive effect of using this biostimulant on the content and activity of some bioactive compounds, such as phenolics and flavonoids, and on the reducing power has also been found in the study. However, further research is needed to understand the mechanisms of action of biostimulants based on seaweed extracts and amino acid on crop plants.

## Author contributions

SK: conceived and supervised the whole study; AS: conceived the study, interpreted data and contributed to the drafting of the manuscript; AK: carried out the field experiment, wrote the manuscript; EC: analyzed the plant material, wrote the manuscript; AW: analyzed the plant material; UB-M: performed the statistical analysis; MK: carried out the field experiment; PF: gave experimental advice and contributed to the drafting of the manuscript. All the authors read and approved the final manuscript.

### Conflict of interest statement

The authors declare that the research was conducted in the absence of any commercial or financial relationships that could be construed as a potential conflict of interest.
